# GraphReco: Probabilistic Structure Recognition for Chemical Molecules

**DOI:** 10.1002/open.202500537

**Published:** 2026-01-29

**Authors:** Haidong Wang, Yuncheng Yu, Jyh‐Charn Liu

**Affiliations:** ^1^ Department of Computer Science & Engineering Texas A&M University College Station Texas USA

**Keywords:** cheminformatics, content extraction, Markov network, optical chemical structure recognition, probabilistic graph model

## Abstract

In scientific documents, molecule structure information is usually delivered by chemical structure images. Although convenient for human interpretation, the image‐based molecule structure depiction is not a machine‐readable format, blocking productivity improvements in many fields including chemical data mining and drug discovery. Molecule structures can be modeled as graphs with atoms as nodes and bonds as edges. Following this intuitive modeling, optical chemical structure recognition (OCSR) can be achieved by first detecting individual atoms and bonds and then assembling into a graph. However, the challenges in decision ambiguity due to false positives and spatial proximity during graph assembly is rarely explained and explicitly addressed. In this work, we present a rule‐based probabilistic OCSR model to explain and tackle the ambiguity challenges in graph assembly. We developed a novel line detection algorithm for precise bond line identification, and designed a candidate mechanism with probabilistic graph to resolve atom/bond ambiguity. The proposed model is evaluated with popular large image datasets and achieved outperforming recognition accuracy compared to state‐of‐the‐art solutions.

## Graphical abstract

Molecule structure images are unfriendly for machine understanding, blocking productivity improvements in chemical data mining, drug discovery, and many other fields. We present a rule‐based probabilistic Optical Chemical Structure Recognition model to explain and tackle the ambiguity challenges in graph assembly. We developed a novel line detection algorithm for precise bond line identification and designed a candidate mechanism with probabilistic graph to resolve atom/bond decision ambiguity during graph reconstruction. The proposed model is evaluated with popular large image datasets and achieved outperforming recognition accuracy compared with state‐of‐the‐art solutions.

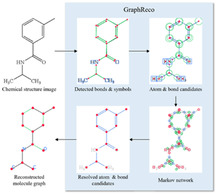

## 1 Introduction

Scientific documents usually contain large amounts of domain knowledge and information. In the chemistry domain, molecule structure plays an important role in conveying composition and spatial arrangement information [1]. Chemical structure diagrams, where atoms are represented by chemical symbols and bonds typically represented by line segments, are widely used in chemistry documents to describe molecule structures. Although some publications have been digitized, a lot of them are still not available in structured formats. By converting molecule structure images into machine‐readable formats, researchers can streamline data mining processes and make the knowledge buried in history documents accessible to the wide public.

However, chemical information encoded in the human‐oriented visual representation is not friendly for machine understanding. First, the same spatial structure of a molecule can have multiple different 2D structure diagrams from different projection perspectives and notation variations of atoms and substructures. Second, structure recognition requires correct identification of every atom and bond, and there might be hundreds of them in a single molecule. And finally, images of chemical structures can be noisy due to annotation marks and low scanning quality. These challenges block potential productivity improvements in many fields including chemical informatics, drug discovery, and pharmacology. The automated conversion from chemical structure images to machine‐readable formats is known as Optical Chemical Structure Recognition.

Existing OCSR solutions generally follow either a rule‐based or a machine learning approach. The earliest OCSR works [2, 3, 4] came out in early 1990s, represented by Kekulé [4]. In 2009, OSRA [5] was published as the first open‐source OCSR solution, and it is followed by more recent works like Imago [6] and MolVec [7]. These works all adopt the rule‐based approach, which recognizes individual molecule components with expert rules and reconstructs each molecule as a graph where atoms are nodes and bonds are edges. The rule‐based approach is featured for its high interpretability and flexibility with domain knowledge integration, but might also be limited in generalization ability.

In recent years, advancement of deep learning has led to the transition to machine learning‐based OCSR solutions. Some of these model‐based systems [8, 9, 10, 11] continue to take advantage of the graph modeling of molecules, identify location and type of atoms and bonds via deep neural networks, and reconstruct the molecule graph. The other model‐based systems [12, 13, 14] adopt the image captioning approach and convert chemical structure images directly to string‐based molecule representations like Simplified Molecular Input Line Entry System (SMILES) [15] and International Chemical Identifier (InChI) [16]. These models use an encoder to encode visual features and then feed to a decoder to generate sequence output. Compared with rule‐based systems, model‐based systems are often better at generalization and noise handling. Yet they also require a much larger amount of training data and computation resources, and their generalization ability also highly depends on pattern presence in training data.

Despite depiction variations, chemical structure images are relatively sparse in general and suitable for variant enumeration. Rule‐based solutions take advantage of expert knowledge, enabling precise control over each decision from bond identification to atom connection. Their behaviors are transparent, interpretable, and tunable and require less data and computation resources than deep learning solutions. These considerations underscore the value of a rule‐based OCSR solution, as presented in this work. However, despite the similarity in processing steps in most rule‐based OCSR algorithms, there still exist hidden and rarely elaborated challenges in this direction. One of them is the precise identification of line segments in bonds, where a small deviation in line ending may break a local atom connection and thus the correctness of the entire structure. The other challenge rarely explained is the decision ambiguity in molecule graph assembly due to false positives and spatial proximity. Spurious atoms or bonds may incorrectly appear as valid, and closely located atoms/bonds might in fact represent the same entity. The system must decide whether to keep, discard, and merge the candidate atoms/bonds, requiring both spatial and structural context to make probabilistic decisions in graph reconstruction.

In this study, we present GraphReco, a knowledge‐driven model for the optical recognition of chemical structure images, to tackle these challenges. We propose a novel line detection algorithm for precise bond line detection and a probabilistic graph model to resolve decision ambiguities in graph assembly. The workflow of GraphReco can be divided into three stages. First, we extract image components of circles, bonds, and chemical symbol shapes. In this stage, we developed an adaptive line detection algorithm to achieve accurate line detection in images of variable resolutions. Then in the second stage, we create candidates for atoms and bonds, infer their most likely state with a probabilistic graph built with Markov network, and resolve atom/bond candidates with inference results. Compared with traditional measures of rigid rules or simple post‐processing, our probabilistic atom/bond candidate mechanism can intelligently handle uncertainty in assembly decisions. It is proved to be able to significantly reduce errors due to noisy and ambiguous input, and it can be beneficial for all OCSR works following the graph reconstruction direction. Finally, we assemble the resolved atoms and bonds as a molecule graph and export it in standard chemical data exchange format.

## 2 Related Works

Over the past three decades, there have been significant research efforts contributing to the field of optical chemical structure recognition. In general, the methodologies fall into two main categories: (1) rule‐based systems and (2) model‐based systems.

Early OCSR systems primarily followed the rule‐based approach, which employed handcrafted heuristics to reconstruct chemical structures as graphs. Users manually define templates and geometric heuristics to identify bonds, atoms, and molecular arrangements. The first complete functional OCSR tool, Kekulé, was released in 1992 [4]. Kekulé employed a rule‐based approach to reconstruct chemical structures from scanned images. The tool included scanning, vectorization, character recognition, graph complication, and post‐processing. It not only identifies atoms and bonds by treating OCR‐resolved characters and image vectors as graph nodes and edges, but also handles stereochemistry and superatoms based on heuristic rules. The system provides users with a graphical interface to inspect, correct, and refine the recognized structures. Optical recognition of chemical graphics (OROCS) [17] and Chemical Literature Data Extraction (CLiDE) [18] later refined this approach by introducing more sophisticated vectorization algorithms and improved heuristics for identifying bond types, atom labels, and stereochemistry. OSRA [5] further improved rule‐based approaches by automating the entire recognition pipeline with OCR engines and batch processing, which can be used to process large‐scale document extraction tasks. ChemReader [19] and MolRec [20] focused on enhancing character recognition and line detection, implementing domain rules to resolve chemical ambiguities and provide improvements to the treatment of overlapping graphical elements and superatoms. Imago [6] offered a modular rule‐based architecture that focused on extensibility and flexibility, providing the system to adapt to various document styles. Later systems such as ChemOCR [21] and Cheminfty [22] incorporated machine learning elements (e.g., OCR components) and a rule‐based backbone for structure parsing and graph assembly.

In recent years, advancements in machine learning have driven progress in OCSR research, particularly toward model‐based approaches. The approach can be categorized into two types: image‐captioning‐based and graph‐construction‐based techniques. For image captioning methods, it frames OCSR as a image‐to‐sequence task which utilizes deep neural networks to extract visual features from chemical structure graphs and decode them into sequential representations, such as SMILES [15] and InChI [16]. The MSE‐DUDL [23] applied a convolutional neural network (CNN) based U‐Net for image segmentation, followed by a CNN+ Recurrent Neural Network (RNN) encoder‐decoder network to produce SMILES output. Later Img2 Mol [24] employed a custom CNN plus a pretrained RNN decoder to convert chemical structure images into SMILES. Then DECIMER 1.0 [12] and Image2SMILES [14] started to use transformer‐based decoder, instead of RNN‐based, with CNN encoder. The introduction of transformer architecture yields high recognition accuracy comparable to traditional rule‐based systems. SwinOCSR [25] applied a Swin Transfromer [26] backbone to capture image features and used focal loss to tackle the token imbalance problem. The image captioning approach also has its limitations. The same molecule may have multiple valid linear representations. They may also encounter difficulty in case of large complex molecule images.

On the other hand, graph reconstruction‐based models consider the intrinsic graph nature of chemical molecules, just like traditional rule‐based works. These models typically identify chemical bonds and atoms with neural networks and directly reconstruct the chemical structure graph. For example, ChemGrapher [8] used a segmentation model followed by three classification networks to identify atoms, bonds, and charges. MolMiner [9] applies a YOLO [27] model to detect atoms and bonds and then assemble them into a molecule graph. MolGrapher [11] first detects candidate atoms using ResNet [28], then classifies atom candidates and bond types with a Graph Neural Network (GNN), and assembles the results as the final graph. The graph reconstruction approach takes advantage of the graph modeling of chemical molecules, allowing for easier integration of domain knowledge and rules.

## 3 System Model

We propose GraphReco to convert 2D chemical structure images into machine‐readable formats. A molecule is a set of atoms connected by attractive forces, known as chemical bonds. Following the graph reconstruction approach, GraphReco models a molecule as a graph with atoms as nodes and bonds as edges and restores the molecular structure by identifying and linking atoms and bonds.

**FIGURE 1:**
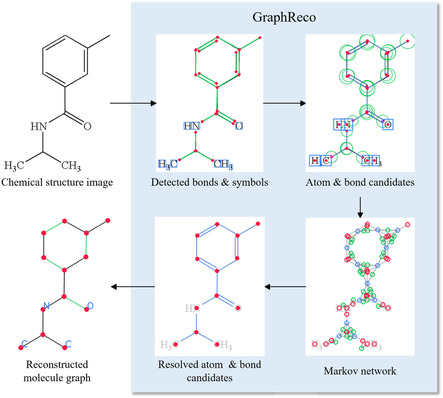
Example of GraphReco molecule recognition process. Given a molecule image, GraphReco first detects bonds and recognizes symbols, then creates candidates for atoms and bonds, and then builds a Markov network to determine the existence and merging state of candidates; then, atom and bond candidates are selected and merged, and finally the molecule is reconstructed as a graph.

Figure 1 shows an example of molecule structure images. In this figure the letters (C, O, N, and H) are element names of carbon, oxygen, nitrogen, and hydrogen, and the subscript numbers indicate the duplication of hydrogen atoms. Atom N and Cs all have close H atoms spatially, indicating that they are all connected with neighboring hydrogen atoms by implicit bonds. The letter atoms connected together form a block, and we call it superatom for simplicity. Besides letters and numbers, there are also many line segments in this figure. These line segments are the typical depiction of chemical bonds in chemical structure images. Each line segment represents one bond between a pair of atoms, whose element name is decided by the situation around the line endings. If a line segment points to a letter atom close enough spatially, like atom N in the figure, then this side of the bond is connected to a nitrogen atom. If there is no letter atom near the line ending, like an open ending or a meeting point of multiple line segments, and then this side of the bond is considered connected to an implicit carbon atom, according to chemistry knowledge. A single chemical bond indicates a pair of electrons participating in the attractive force, each from one atom in the pair, and this is reflected as individual line segments in Figure 1. There are also cases where multiple pairs of electrons form one bond, and they are called double bonds, triple bonds, etc. We call them multibond for short. These multi bonds are represented by multiple parallel and close line segments of similar length. In Figure 1, we have four double bonds, three in a ring, and one connected to the oxygen atom.

Semantically, a molecule consists of atoms who are connected by covalent bonds (ions ignored). These semantic notions are expressed by geometrical shapes in images. Grayscale images are composed of pixels with 2D coordinates, and a pixel has its 4‐neighbors and 8‐neighbors whose x/y coordinate difference with the target pixel is exactly 1. In this paper we use 8‐neighbor as the definition of neighbors. In a binary image of white background, we can define a *pixel path* as a set of pixels where there is either one single black pixel or each pixel has at least one neighbor in the same set. And then a shape can be defined as a set of pixels where there is always a path of black pixels between two different pixels in this set.

When a molecule structure is depicted in an image, atoms are denoted by either English letters explicitly or bond endings implicitly (carbon only). Compared with simpler single atoms, superatoms as a block of markup text may contain letters, superscript of charges (“+”/“−” sign, optionally numbered), subscript of duplications, and brackets for grouping. Besides combinations of marked‐up element names, superatoms may also appear as shorthand notations without valid element names, like “Me” and “OTos.” To make a difference with bond shapes, we use the term *symbols* at semantic level to represent these pixel‐level shapes, including all the shapes that's typically perceived by humans as letters, numbers, +/− signs, and brackets.

**FIGURE 2:**
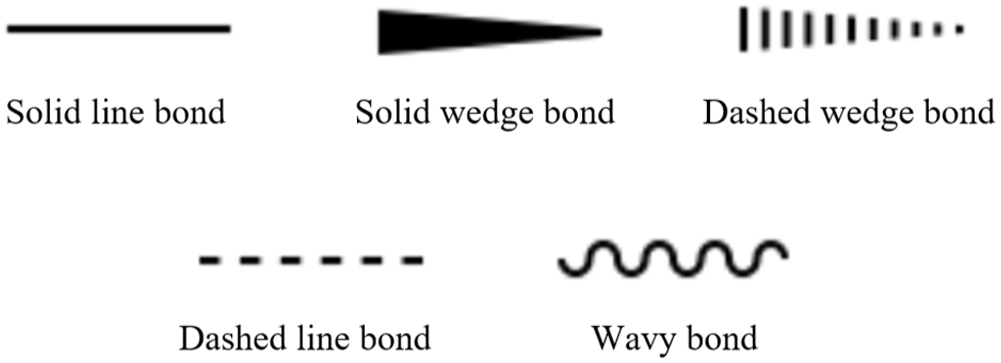
Common styles of chemical bond depictions in chemical structure images. From left to right, top to down, they are solid line bond, solid wedge bond, dashed wedge bond, dashed line bond, and wavy bond.

As connections between atoms, bonds are usually depicted by solid line segments. Mathematically, a line segment is a straight line of finite length connecting two distinct points. In binary images of white background, theoretical line segments are expressed with a black thin shape stretching along one direction, and coordinate of each point on the line is rounded to integers. There are also other less common styles of bond depictions, including solid wedge, dashed line, and wavy curves, as shown in Figure 2. Single‐headed arrows are ignored considering that they usually don’t represent a single bond. Besides bonds, symbols, and background pixels, there might also be noise in an image of chemical structure. We assume the input of our system is an image containing one 2D molecule structure and possible noise. The noise includes nonbackground pixels unrelated to the integrity of molecule structure, usually captions and other explanatory marks.

**FIGURE 3:**
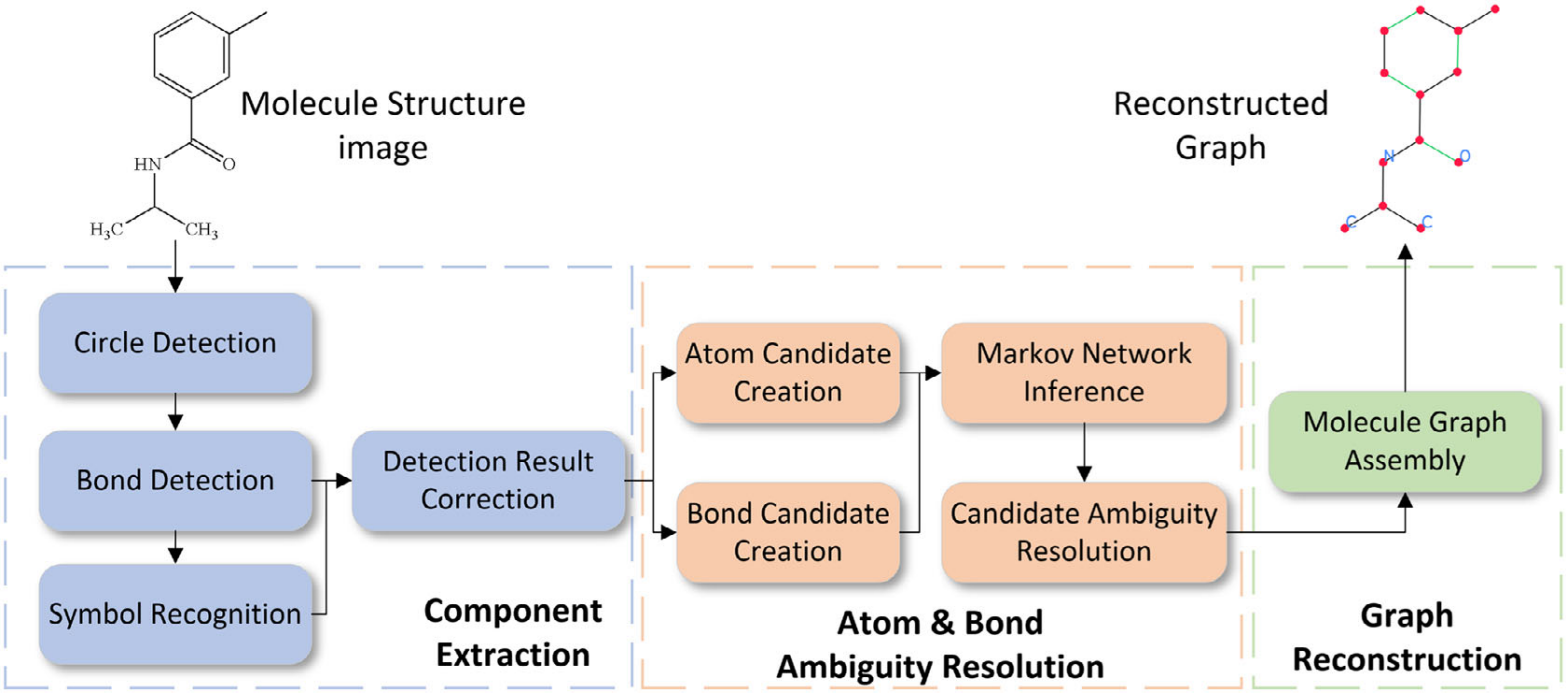
Overall architecture of the proposed GraphReco system.

The overall architecture of GraphReco is illustrated in Figure 3. The workflow can be divided into three stages. In the first stage, individual shapes are extracted: circles, bond shapes, and chemical symbol shapes. Then, in the second stage, we create atom and bond candidates and resolve their states with Markov network. And finally, candidates are resolved to final atoms and bonds, and we assemble them into a molecule graph.

## 4 Component Extraction

In order to reconstruct the molecule as a graph, it is necessary to first extract individual bonds and atoms. The skeleton of molecules is built by chemical bonds. So in GraphReco, bonds are detected and masked before locating atoms/superatoms. Most bonds are formed by line segments, which can be handled by line detection algorithms. Sometimes, there are also circles in the input images, as an integral part of aromatic bonds. Circular curves, especially those with small curvature, are geometrically similar to lines. To avoid interference with bond line detection, we first apply preprocessing, then detect and mask long circular curves before bond detection.

### 4.1 Circle Detection

Given an image of 2D chemical structure, GraphReco first applies preprocessing operations. If the image is not binary, it is converted to binary image with white background. Then, the white background borders are trimmed to remove large white spaces. After this, the image is padded, upscaled (if too small), and thinned with the Zhang–Suen thinning algorithm [29] to eliminate the impact of shape width on bond line detection and further operations.

With the preprocessed image, we apply the circle Hough transform [30] (CHT) to find out circular curves with radian greater than 180°, including circles. The CHT algorithm detects circular curves by finding voting peaks in the circle parameter space. A circle centered at (a,b) with radius r can be defined as

(1) $${\left(x-a\right)}^{2}+{\left(y-b\right)}^{2}=r^{2}$$

where (x,y) are points on the circle. Then, with a list of r values, each point (x,y) leads to an array of possible circle centers (a,b), and thus adding a vote to point (a,b,r) in the parameter space. After enumeration of all the allowed r values, restricted by image size, local peaks will emerge, suggesting centers and radii of circles. These local peaks are then filtered by nonmaximum suppression to produce parameters of detected circles. CHT works in the parameter space instead of the image space, so it can also detect circular curves as long as they are long enough to yield a peak in the parameter space.

### 4.2 Bond Detection

#### 4.2.1 Solid Line Bonds

In 2D chemical structure images, bonds are typically depicted with a line segment or multiple parallel line segments of similar length, as shown in Figure 1. This makes it possible to recognize the majority of bonds by detecting line segments. Hough transform [31] is a classical method to detect lines in images. Like CHT, Line Hough Transform (LHT) also works by finding voting peaks in the parameter space. The difference is that in line detection the target shape, line, is defined as

(2) r= x⋅cos(θ)+y⋅sin(θ)

where r is the distance between the origin and the closest point on line, θ is the angle between *x* axis and the line from origin to the closest point on line, and (x,y) is a point on the input image. Like CHT described above, LHT is resistant to broken lines of multiple segments.

However, line segments in structure images often suffer from severe pixelization, especially in small images. On the other hand, the detection of bond lines requires high accuracy in locating the line endings, as the line endings are directly related to correct connection with atoms. In LHT, some algorithm parameters are closely related to detection result and computation efficiency, including resolutions for r and θ, and minimal line length. If these resolution thresholds are large, short lines and lines close to each other may be missed. If they are very small to obtain finer results, especially when smaller than 1 pixel and 1°, many overlapping line fragments covering part of actual bond lines may be returned. It is difficult for classical LHT to deliver satisfactory performance in bond line detection when dealing with images of variable sizes, where line length and distance vary significantly. Therefore, we developed the *Fragment Merging* (FM) algorithm, an adaptive three‐stage line detection algorithm based on LHT, to achieve accurate detection of chemical bond lines.

In the first stage of the FM algorithm, we apply classical LHT on thinned image. The resolutions for r and θ in this LHT are relatively small ($\text{resolutio}n_{r}=2,\text{resolutio}n_{\theta }=2\deg$) to fit for short and close lines. Then, we walk along the detected theoretical lines from LHT, find black pixels on the same mathematical line, group them by connectivity, and obtain the endings of each pixel group. Each pixel group forms a line fragment, usually shorter than the corresponding bond line it's on. This is the vectorization of the line fragments.

In the second stage, we group and merge these line fragments. Line fragments on different real bond lines exhibit important differences, enabling us to classify them into different groups. Two line fragments on the same bond line usually have very similar angles. If two line fragments are not close from each other in the direction perpendicular to them, they are unlikely to be on the same line. If these two criteria are both satisfied, we can check their overlapping status. The two line fragments are supposed to be on the same bond line if they are overlapped or there exists a path of black pixels between them. Detection of the black pixel path is achieved by sampling in the space between their nearest line ending points.

Finally in the last stage, line fragments belonging to the same group are merged into one line segment as detection result. Two border pixels farthest from the centroid of the pixel set are selected as ending points of the merged line segment. The workflow of the FM line detection algorithm is described with pseudo code in Algorithm 1. The two grouping functions, group_by_px_connectivity() and group_frag_pairs(), work in a similar way, and they can both be implemented with the union find algorithm [32]. In the scenario of bond line detection, statistics of the final merged line fragments are computed and those shorter than 1/10 of average line lengths are discarded, because bond lines in the same structure image are usually uniform in length distribution.

ALGORITHM 1 **The fragment merging algorithm for line detection.**

**FIGURE 4:**
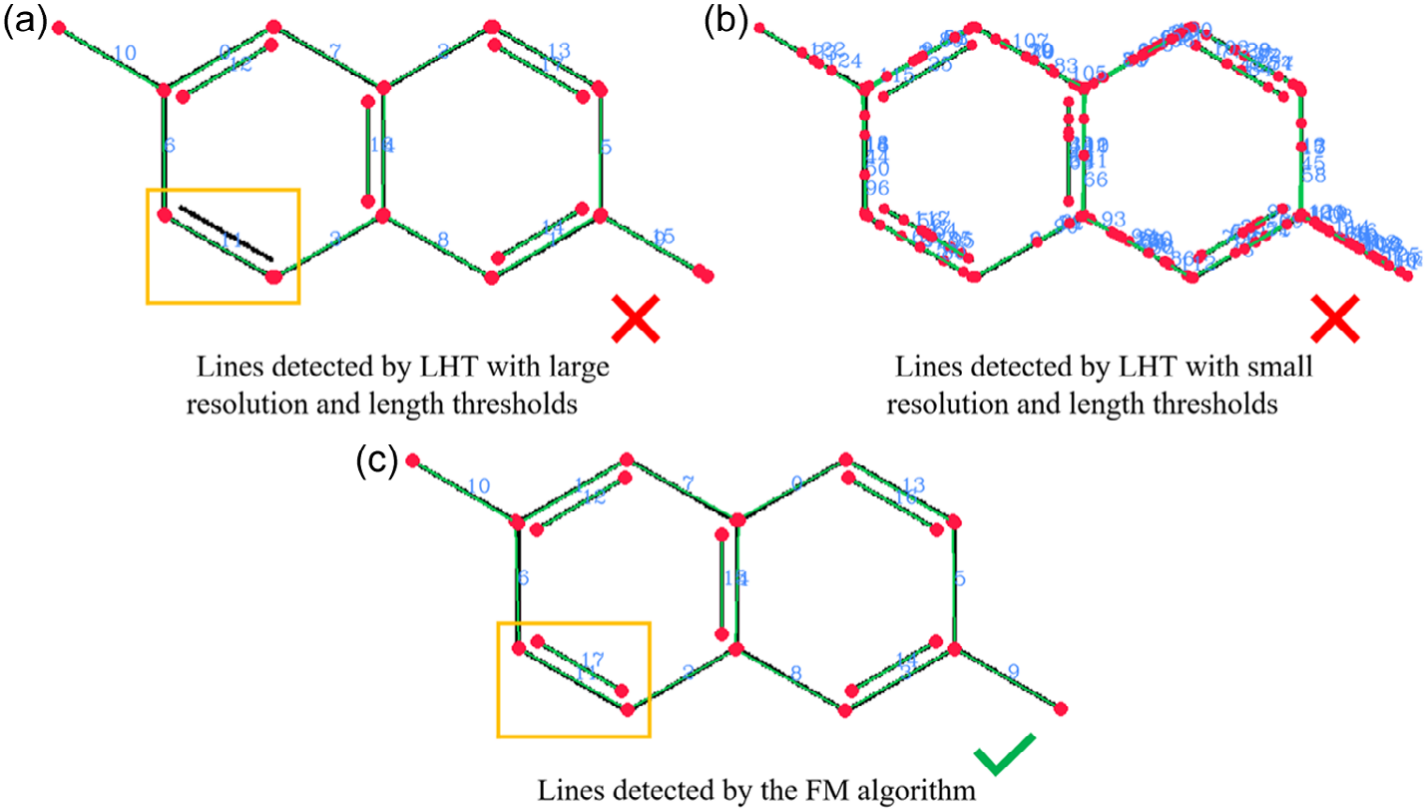
Comparison of line detection results by LHT with different parameters and the proposed FM algorithm. The original image is binary with a black shape, the red dots denote line ending, the green pixels denote the pixels on the detected lines, and the blue numbers are labels for detected lines.

1 000001  1: function **fragment_merging**(img):000002  2:*  // Stage 1: obtain line fragments*000003  3:  rhos, thetas = **line_hough_transform**(img);000004  4:  black_px_set = set();000005  5:  for rho, theta in zip(rhos, thetas):000006  6:   for x in range(0, img_width): // iterate over y if theta > 45 deg000007  7:    y = int(rho/sin(theta) – x/tan(theta));000008  8:    for y_opt in range(y‐search_range, y + search_range + 1):000009  9:     if img[x, y_opt] is black:000010  10:     black_px_set.add((x, y_candidate));000011  11:  line_fragment_px_groups = **group_by_px_connectivity**(black_px_set);000012  12:  line_fragments = **get_line_endings**(line_fragment_px_groups);000013  13:000014  14:*  // Stage 2: group line fragments*000015  15:  frag_pairs_set = set();000016  16:  for frag1 in line_fragments:000017  17:   for frag2 in line_fragments:000018  18:   if frag1 == frag2 or **has_checked**(frag1, frag2):000019  19:    continue;000020  20:   if **angle_difference**(frag1, frag2) > angle_threshold:000021  21:    continue;000022  22:   dist_1to2 = pt_distance_to_line(frag1.center, frag2);000023  23:   dist_2to1 = pt_distance_to_line(frag2.center, frag1);000024  24:   if min(dist_1to2, dist_2to1) > dist_threshold:000025  25:    continue;000026  26:   if **is_overlapped**(frag1, frag2) or **has_black_path_between**(frag1, frag2):000027  27:    frag_pair_set.add((frag1, frag2))000028  28:000029  29:*  // Stage 3: merge grouped line fragments*000030  30:  line_fragment_groups = **group_frag_pairs**(frag_pair_set);000031  31:  final_lines = set();000032  32:  for frags in line_fragment_groups:000033  33:  if x_range(frags) > y_range(frags):000034  34:   line = Line(**leftmost_end**(frags), **rightmost_end**(frags));000035  35:  else:000036  36:   line = Line(**topmost_end**(frags), **bottommost_end**(frags));000037  37:  final_lines.add(line);000038  38:  return final_lines;

A comparison of line detection results by LHT and the FM algorithm is shown in Figure 4. When applied with coarse resolutions for distance/angle resolution and line length, there can be undetected lines and overlapping lines. As shown in Figure 4a, the bottom left backslash line segment is not detected, and the bottom right backslash line segment yields two overlapping detected lines. In Figure 4b are the small line fragments detected by LHT with high‐resolution parameters. Although in this case all the bond lines are covered, we can see the results are all fragments shorter than the actual bond line. The detection result of the FM algorithm is shown in Figure 4c, where every bond line is correctly identified. The FM algorithm is able to recognize line segments of different angles and distances accurately, adaptive to line segments of varying length and distance to each other.

The proposed FM line detection algorithm starts with an LHT with high resolution parameters, enabling it to detect short lines and lines of varying angles. The false peaks introduced by high‐resolution parameters is eliminated by the subsequent fragment merging process. In the first stage of the algorithm where alignment of mathematical line and image shape happens, we search for black pixels in a small area around the theoretical line point (a window of 5 pixels is applied for each x/y coordinate), allowing for some extent of pixelization. In the second stage, the sampling of black pixel paths between non‐overlapped line fragments further allows for small discontinuity in line pixels. Therefore, the FM algorithm is robust in the detection of lines in chemical structure images of variable sizes. However, images suffering from severe pixelization and noise may result in distorted lines after thinning and large ruptures, and in these cases, the FM algorithm might also fail in correct line detection. Therefore, image preprocessing is also essential for accurate bond detection.

The proposed FM algorithm can detect line segments in solid line bonds with one or more line segments. For multibonds like double and triple bonds, they have multiple parallel solid line segments of similar length and high overlapping ratio along the line direction. When there are multiple line segments whose angles and distances are very close, and they have a high overlapping ratio over either of them, they’re combined into one multibond (double or triple, depending on line segment number). For single bonds, the bond ending points are the same as the ending points of the corresponding line segment. For multibonds, the average point of line endings at the same side of bond is taken as the corresponding bond ending. Occasionally, two long line segments intersect each other, indicating an implicit carbon atom at the intersection point. In this case, the long line segments are split at the intersection point into separate shorter bonds. There are also cases where one single solid line bond is connected to a double/triple solid line bond, and they share exactly the same direction. They appear as a longer line segment and a parallel shorter one close to the long one. In this case, the longer line segment is split at the point closest to the ending of the shorter line segment for bond creation.

#### 4.2.2 Nonsolid‐Line Bonds

Besides solid‐line segments, chemical bonds may also be depicted with other shape styles, typically in the form of solid wedges, dashed wedges, dashed lines, and wavy curves as in Figure 2. We also developed detectors for each of these bond styles. Solid wedges are transformed into thin lines after the thinning preprocessing. So we first detect a solid wedge bond as solid line single bond and then go back to the binary image before thinning to extract the width profile of a strip along the line. The width profile is then analyzed to check whether it appears as a wedge shape, as explained in detail in Algorithm 2. In terms of dashed line bond and dashed wedge bond, they both consist of parallel overlapping short line segments close to each other. So we find such short line segments from thin shapes and create dashed bonds from them, as described in Algorithm 3. In this process, dashed line and wedge bonds are differentiated by the length variance of the short line segments. We also developed a detection algorithm for wavy bonds, as shown in Algorithm 4. After identification of solid line bonds, wavy bonds are isolated into shapes with consecutive interleaved circular curves. So we leverage this feature, detect long circular curves from individual symbols, filter out symbols with unqualified curves, and create a wavy bond from the symbol. In terms of aromatic bonds, besides the regular interleaved single and double bond style, sometimes they are also depicted as single‐bond polygons with a circle inside. Such aromatic bond styles are identified with the help of previously detected circles. Illustration of bonds with different styles, together with their successful detection, are provided in the experiment section below.

ALGORITHM 2 **Solid wedge bond detection.**

1 1: function **detect_solid_wedge_bond**(bonds, img_bin):2:  for bond in bonds:3:   If bond.degree != 1:4:    continue;5:   line = bond.lines [0];6:   sample_pts = **sample_points_along_line**(bond.lines [0], num_samples);7:   sample_pts = **keep_center_fraction**(sample_pts); // avoid edge effect8:   widths = **compute_shape_width_along_strip**(sample_pts, line);10:   start, end = widths [0], widths[‐1];11:   if max(start, end) < min_max_width:12:   continue;13:   ratio = abs(start – end) / min(start, end);14:   if ratio >= min_wedge_ratio:15:   bond.is_solid_wedge_bond = True;16:  return;

ALGORITHM 3 **Dashed bond detection.**

1 1: function **detect_dashed_bond**(bonds, symbols, thinned_img_shapes):2: // collect thin shapes3: thin_shapes = set();4: for shape in thinned_img_shapes:5:  max_wh = max(shape.width, shape.height);6:  if is_thin_shape(shape) and max_wh <= max_len:7:   thin_shapes.add(shape);8:9: // group thin shapes parallel and close to each other10: for i in range(0, len(thin_shapes)):11:  shape1 = thin_shapes[i];12:  for j in range(*i* + 1, len(thin_shapes)):13:   shape2 = thin_shape[j];14:   dist = **get_distance**(shape1.center, shape2.center);15:   angle = **get_angle**(shape1, shape2);16:   if dist <= max_dist and angle <= max_angle:17:    shape1.neighbor_shapes.add(shape2);18: shape_groups = **group_shapes_with_union_find**(thin_shapes);19:20: // create dashed bond from thin shape groups21: for shapes in shape_groups:22:  // skip groups with too few shapes23:  if len(shapes) < 4:24:   continue;25:  if var([s.length for s in shapes]) < len_var_threshold:26:   dashed_bond = **DashedLineBond**(shapes);27:  else:28:   dashed_bond = **DashedWedgeBond**(shapes);29:  bonds.add(dashed_bond);30:  bonds.remove(**get_on_shape_bonds**(bonds, shapes));31:  symbols.remove(**get_on_shape_symbols**(symbols, shapes));

ALGORITHM 4 **Wavy bond detection.**

1 1: function **detect_wavy_bond**(bonds, symbols, median_bond_len):2: // collect candidate symbols for wavy bond3: candidate_symbols = set();4: for symbol in symbols:5:  w, h = **get_min_area_rectangle_info**(symbol);6:  if max(w, h) < median_bond_len/2 or min(w, h) > median_bond_len/2:7:   Continue;8:9: for symbol in candidate_symbols:10:  // detect circles from symbol pixels11:  curves = **detect_long_circular_curves**(symbol.pixels);12:13:  // if too few curves on symbol, skip14:  if len(curves) < min_num_circles:15:   continue;16:  // if circle centers are not very likely on a line, skip17:  center_variance = **fit_circle_centers_to_line**(curves);18:  if center_variance > max([c.radius for c in curves]):19:   continue;20:  // if curvature variance is too large, skip21:  curvatures = [c.curvature for c in curves];22:  if avg(curvatures) < Pi or var(curvatures) > max_curvature_var:23:   continue;24:25:  // create wavy bond and remove corresponding symbol26:  wavy_bond = **WavyBond**(symbol, curves);27:  bonds.add(wavy_bond);28:  symbols.remove(symbol);

### 4.3 Symbol Recognition

A chemical structure image consists of pixels from bonds, symbols, noise, and background. With bonds detected and masked, the remaining shapes are considered as symbols. Small noise shapes are filtered out. Individual shapes close to each other, like individual letters and numbers, are grouped together as shape groups (also known as superatoms in some other works). At semantic level, shape groups are groups of symbols. Semantic symbols in a shape group can be classified as superscript, subscript, and mainline symbols, depending on their relative layout inside the group. Superscript symbols include +/− charges and count of charges, subscript symbols contain numbers, and mainline symbols include element names and brackets.

The shapes and shape groups are converted to text by the Tesseract Optical Character Recognition (OCR) engine [33]. Tesseract is a popular open‐source OCR engine with support for more than 100 languages out of the box. Tesseract OCR recognition performs well with nonskewed single‐line and multi‐line properly spaced characters without superscript or subscript. So consecutive symbols in the same category (mainline, superscript, or subscript) are fed as a single block into the OCR engine, and adaptive upscaling, smoothing, and dilation operations are also applied in advance to improve recognition accuracy. A symbol group can be seen as a shorthand notation with internal connections omitted. The internal atom connections are determined by chemical rules. Also from chemistry knowledge we know that hydrogen atoms can be omitted without changing chemical meaning in scenarios of visualization and toolkit handling. So in our system, symbol groups with at most one nonhydrogen atom are recognized, parsed, and converted to atoms directly. More complicated cases are handled by substitution with precomputed substructure dictionary.

### 4.4 Detection Result Correction

It is difficult to achieve perfect extraction of bonds and symbols. In order to provide more accurate bonds and symbols for the assembly of the molecule, it is necessary to correct the false positives of bonds and symbols, as a semi‐open‐loop correction of the extraction process. Pixels on the input image fall into the categories of bond, symbol, noise, and background. Small noise shapes are discarded during the symbol extraction. Larger noise shapes like captions might be comparable to atom symbols in terms of size, and they will be filtered out in the final assembly phase. So we focus on two cases here: 1) whole or part of symbol classified as bond and 2) whole of part of bond taken as symbol.

Some shapes, like shapes of letter H and N, contain linear parts which are often recognized as bond line segments. If it is not a thin shape, then we use OCR result to determine whether the shape can be a valid chemical symbol. Things are trickier for thin shapes of very small width or height (regardless of rotation). These thin shapes could be either single bonds, part of multibonds, or chemical symbols like letter l in Cl and letter I for Iodine, as enumerated in [34]. In this case, if the shape is contained in a multibond or pointing to at least one other symbol, then it is a valid bond. Otherwise, the shape is considered a symbol. Symbol false positives are less common than bond false positives. We only consider the case where solid line bonds are taken as symbols by mistake, as bonds of other styles have been detected separately. Solid line bonds are composed of line segments, so thin shapes that are neither horizontal nor vertical are restored as bonds.

The false‐positive correction measures form a semi‐open‐loop correction for the detection stage. Correction of bond false positives improved the recognition of letters like H and N, which are among the most common elements in chemical molecules. Correction of symbol false positives successfully restored relationship between neighboring atoms. Together they effectively improved the detection results of both bonds and symbols in empirical experiments.

## 5 Atom and Bond Ambiguity Resolution

### 5.1 Atom and Bond Candidate Creation

With detected bonds and recognized symbol groups, we are able to construct molecule graphs. Each bond ending point indicates an atom. If there are symbols in the vicinity of the bond ending, the element name of the indicated atom is decided by the symbols, which can be obtained from OCR results. In case of no symbols around bond ending point, the atom is an implicit carbon atom according to chemistry knowledge. Two atoms connected to the same bond are considered connected. Then, we can build a graph where atoms are nodes and bonds are edges, by creating atoms and linking them with bonds of corresponding types. This is an intuitive approach to reconstruct the molecule graph.

However, the recognition accuracy of this naive graph assembly method turned out to be very low, because lower‐level component extraction results are often not precise enough, resulting in significant ambiguity in assembly decisions. For example, at furcation points where multiple bonds meet at one implicit carbon atom, a small deviation in the ending point location of one bond may lead to wrong connection at this atom. A single small error like this can break the entire graph structure. To tackle this issue, we create atom and bond candidates from lower‐level extraction results and use the probabilistic Markov network to infer the most probable state of the candidates.

The lower‐level component extraction process produces bonds represented by bond endings and symbol groups represented by bounding boxes and OCR results. The bond endings and symbol groups suggest presence of atoms, and the detected bonds suggest presence of chemical bonds. To map these results to the final molecule graph in a probabilistic way, we define and create *atom candidates* and *bond candidates*. Atom candidates are created from bonds and symbol groups, and each atom candidate is assigned a “bounding area” (we use A(a) to refer to the bounding area of atom candidate a) to assist further atom merging and connection. Overlapping of bounding areas from different atom candidates suggests the possibility in them belonging to the same atom. At each bond ending point, an atom candidate is created, with its bounding area defined as a circle of radius $r_{b}$

(3) $$r_{b}=\min\left(l_{\mathrm{bond}},l_{\mathrm{med}}\right)/4$$

where $l_{\mathrm{bond}}$ is the length of the corresponding bond, and $l_{\mathrm{med}}$ is the median length of all detected bonds. At each symbol group, atom candidates are created from the symbols close to bond endings nearby (typically leftmost/rightmost or topmost/bottommost mainline symbols), centering at the symbol bounding box center, with its bounding area defined as a square of side length $sl_{b}$

(4) $$sl_{b}=1.5\times \max\left(sl_{\mathrm{sym}},sl_{\mathrm{med}}\right)$$

(5) $$sl_{\mathrm{sym}}=\max(\text{width},\text{height})$$

where $sl_{\mathrm{sym}}$ is the larger one of width and height of symbol bounding box, and $sl_{\mathrm{med}}$ is the median $sl_{\mathrm{sym}}$ of all detected symbols. Bond candidates, on the other hand, are created from each detected bond with the same ending points. These candidates will be kept, discarded, or merged depending on the probabilistic inference result of the Markov network.

### 5.2 Markov Network Inference

Markov network [35, 36, 37], also known as Markov Random Field, is an undirected probabilistic graph model that defines a joint distribution over a set of random variables $X=(X_{1},X_{2},\ldots ,X_{n})$. Each random variable represents a node in an undirected graph, where the edges represent probabilistic dependencies between nodes. The Markov network defines a non‐negative potential function $\phi_{c}$ for each clique c in the graph, where a clique is a set of fully connected graph nodes. If we use P(X=x) to represent the probability of variable X taking the value x, then the join distribution can be defined as

(6) $$P\left(X=x\right)=\frac{1}{Z}\prod_{c}\phi_{c}(x_{c})$$

where $\phi_{c}(x_{c})$ is the potential function over clique c and Z is the partition function for normalization, defined as $Z=\sum_{x}\prod_{c}\phi_{c}(x_{c})$. The potentials can also be written in exponential form as

(7) $$P\left(X=x\right)=\frac{1}{Z}\exp(\sum_{c}w_{c}f_{c}(x_{c}))$$

where $f_{c}(x_{c})$ is the feature functions as the indicator of configuration in clique c and $w_{c}$ is the corresponding weight. In a Markov network, value of some variables can be observed; these variables are called *evidence*. Feeding observed variables of evidence into a Markov network can reduce the uncertainty of the distribution. In this case, maximum a posteriori (MAP) inference is performed on the remaining variables. Markov network has been employed by previous work to clean up noise in lower‐level detection results [34], but our work utilizes it with new rules and optimization strategies and achieves higher recognition accuracy.

Based on atom and bond candidates, we define four types of graph nodes in the Markov network: atom nodes, bond nodes, atom merge nodes, and bond merge nodes, as explained below:

1. *Atom node*: Boolean variable created for each atom candidate, indicating its existence.
2. *Bond node*: Boolean variable created for a bond candidate, indicating its existence.
3. *Atom merge node*: Boolean variable created for pairs of atom nodes, indicating whether these two atom nodes should be merged into one or not.
4. *Bond merge node*: Boolean variable created for pairs of bond nodes, indicating whether these two bonds should be merged into one or not.

Spatial information from atom and bond candidates are fed into the graph model as observation, including ending points of all bonds (and solid line segments, if exists) and bounding boxes of all the symbol blocks. Atom merge nodes are created for every pair of atom node whose bounding areas overlap. Bond merge nodes are created for every pair of bonds whose Euclidean distance of centers is no greater than the length of the longer bond, and bond center is defined as the average point of bond endings.

After creation of graph nodes, we can define functions, also known as *factors* in Markov network, over the node variables to represent how likely each combination of variables is. These probability scoring functions are created from the rules defined below:

1. An atom candidate $a_{1}$ exists (existence factor $\phi (a_{1})=0.9$) if
  a. It is created from bond ending.
  b. $\exists a_{2},A(a_{1})\cap A(a_{2}) \neq \emptyset$, where $a_{2}$ is another atom candidate created from bond ending. If not created from bond ending, the atom candidate must be created from explicit symbols. This rule requires its bounding area to overlap with that of at least one other atom candidate created from bond ending.
2. A bond candidate b exists (existence factor ϕ(b)=0.9) if
  a. It is not created from solid line segments.
  b. $\max(\mathrm{dist})\leq l_{b}/8$, where dist is the distance of evenly sampled points to their closest black pixel, and $l_{b}$ is the bond length. This requires each of the bond's line segments should be covered by or very close to non‐background pixels.
3. An atom candidate $a_{1}$ is likely to be merged with another atom candidate $a_{2}$ if
  a. Both atom candidates exist: $var(a_{1})=1$ and $var(a_{2})=1$, where $var(a_{i})$ is the existence variable for atom candidate $a_{i}$.
  b. Their bounding areas overlap, and at most one of them is created from symbol group. The closer their centers are, the higher the merging likelihood is, as explained in Equations (8) to (11).
4. A bond candidate $b_{1}$ is likely to be merged with another bond candidate $b_{2}$ if
  a. Both bond candidates exist ($var(b_{1})=1$ and $var(b_{2})=1$).
  b. Both atom candidates created from $b_{1}$ are likely to be merged with those created from $b_{2}$.

If two atom candidates $a_{1}$ and $a_{2}$ both exist and are created from bond endings, then their merging likelihood $P(a_{1},a_{2})$ is defined as

(8) $$P\left(a_{1},a_{2}\right)=\left\{ \begin{array}{c} 0.9,ifd\leq Q\\ 0.7-0.4\left(d-Q\right)/(R-Q),ifQ<d\leq R\\ 0.1,ifd>R \end{array}\right.$$

(9) $$Q=\max\left(r_{1},r_{2}\right),R=\min\;(1.5Q,r_{1}+r_{2})$$

where d is their center distance. If $a_{1}$ is created from bond endings but $a_{2}$ is not, their merging likelihood $P(a_{1},a_{2})$ is defined as

(10) $$P\left(a_{1},a_{2}\right)=\left\{ \begin{array}{c} 0.9,ifd\leq S\;\mathrm{and}\;{\mathrm{line}}_{a_{1}}\cap A_{a_{2}}\neq \emptyset \\ 0.7-0.4\left(d-S\right)/(T-S),ifS\leq d\leq T\\ 0.1,ifd>T \end{array}\right.$$

(11) $$S=sl_{a_{2}}/2+\max\left(r_{a_{1}},l_{\mathrm{med}}/4\right),T=\min(1.5S,sl_{a_{2}}+\max\left(r_{a_{1}},l_{\mathrm{med}}/4\right))$$

where $l_{\mathrm{med}}$ is the median length of detected bonds, $line_{a_{1}}$ is the straight line (of infinite length) specified by the ending points of the bond where $a_{1}$ is created from, and $A_{a_{2}}$ is the bounding area of $a_{2}$. If symbol $a_{2}$ is smaller than median symbol size $sl_{\mathrm{med}}$, $sl_{a_{2}}$ is enlarged to 1.5 times here to better handle distant connections. If an atom candidate created from symbol lies on the direction pointed to by a nearby bond, then it is likely the symbol and the bond are connected.

**FIGURE 5:**
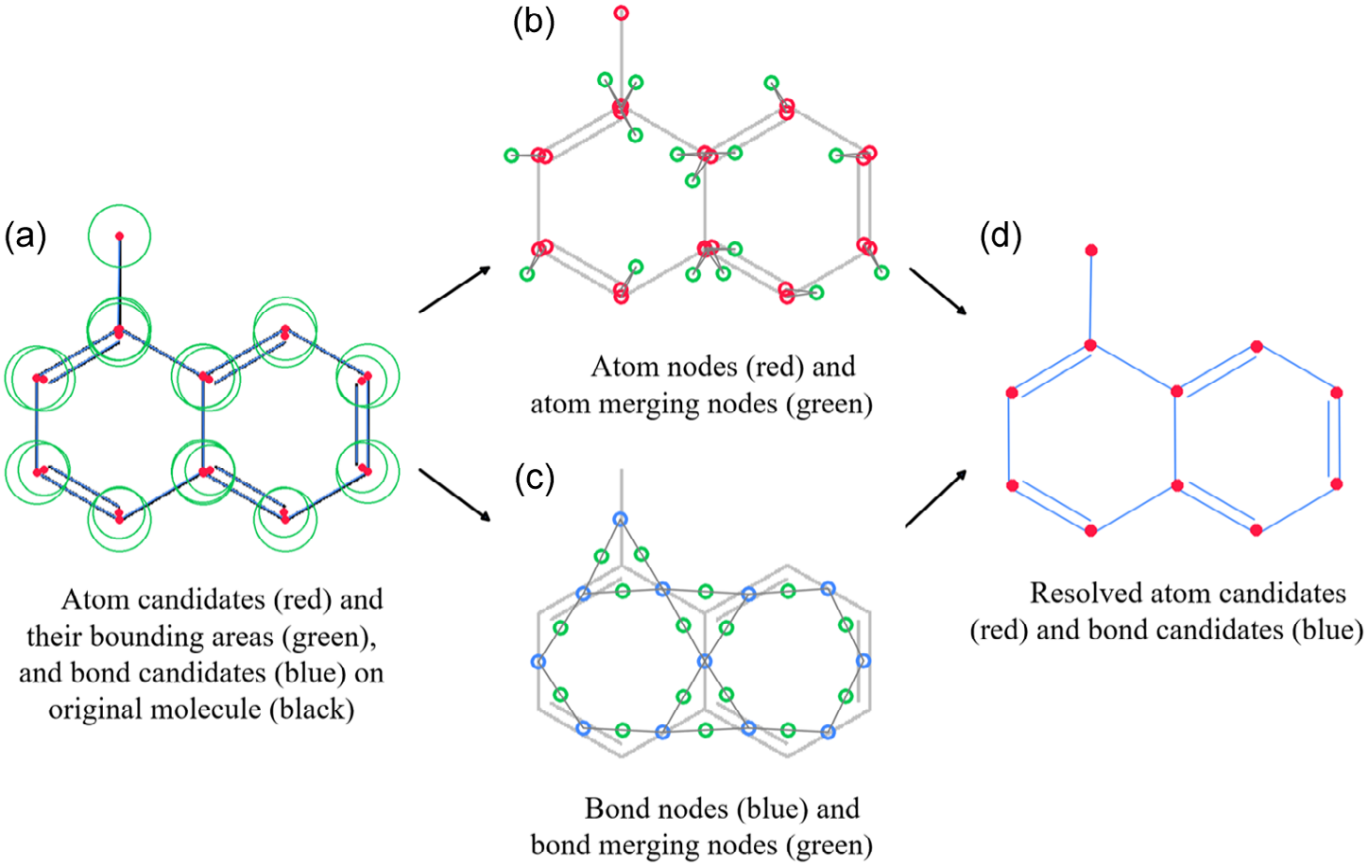
Creation and ambiguity resolution of atom and bond candidates. In subfigure (a), atom and bond candidates are created. Subfigures (b) and (c) are atom/bond nodes and atom/bond merging nodes created in the Markov network. The inference result helps resolve the existence and merging state of atom/bond candidates, as in subfigure (d).

With the nodes and functions defined and observations fed into the graph, we can perform inference to obtain the MAP state of the node variables. The existence and merging decisions obtained will then be used to assemble the final molecule graph, as shown in Figure 5.

### 5.3 Candidate Ambiguity Resolution

From the Markov network output, we can know the atom and bond candidates most likely to exist, and the other ones will be discarded. Then we can merge atom candidates following network output. Atom candidate merging is a growing process. If atom candidate $a_{1}$ should be merged with $a_{2}$, and $a_{2}$ should be merged with $a_{3}$, then all of these three atom candidates are grouped and merged. The final merged atom $a_{\text{merged}}$ is centered at the average center of atom candidates $a_{i}(i=1,2,\ldots ,n)$. Merging of bonds follows the same growing style, and endings of the merged bond are the averaging endings of corresponding bond candidates. One difference in bond merging is that the degree of the merged bond is the accumulation of the candidates. The type of atoms can be determined after merging of candidates. If there are atom candidates created from symbol groups, the element name of the merged atom is decided by symbol group OCR result. Otherwise the merged atom is an implicit carbon atom.

## 6 Graph Reconstruction

Connection relationship between atoms and bonds can be directly known from the candidate merging process. If one of the atom candidates created from endings of bond B is merged into atom A, then bond B is connected to atom A. With bonds as bridge, we can establish connection relationships between atoms. The connected atoms and bonds together form the graph representation of the target molecule. Noise on images may result in multiple smaller graphs created by mistake, so we select the largest one as the final molecule graph. The assembled graph is then exported as an MDL Molfile [38], which is a standard data exchange format for chemical molecule representation. An MDL Molfile contains an atom list, an atom connection table, and other metadata [3]. It is supported by popular open‐source cheminformatics toolkits like RDKit [39] and Chemistry Development Kit (CDK) [40], enabling potential broader applications of molecule recognition results.

## 7 Evaluation

### 7.1 Experiment Setup

**FIGURE 6:**
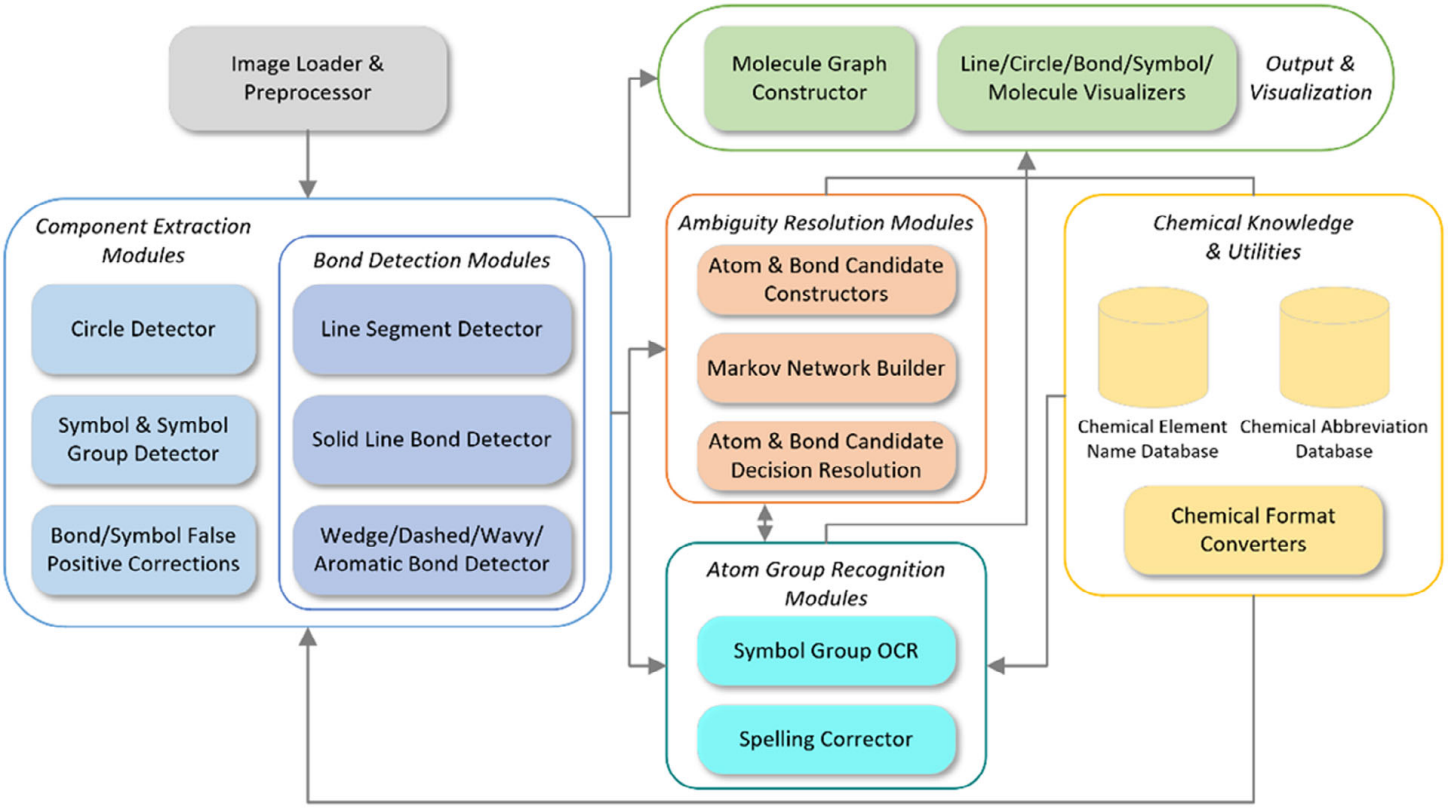
Software architecture of the GraphReco implementation.

We implemented GraphReco in Python, as outlined in Figure 6. The system takes images of 2D chemical structure as input and then detects and extracts circles, bonds, and symbols with component extraction modules. Symbol groups are then recognized and converted into substructures with help from chemical element name and abbreviation databases. The ambiguity resolution subsystem builds a Markov network using the molecule components and determines the most likely atoms and their connection bonds. The result is then used to construct a molecule graph and export to Molfile. In the meantime, intermediate detection and final reconstruction results are visualized with OpenCV and RDKit.

**FIGURE 7:**
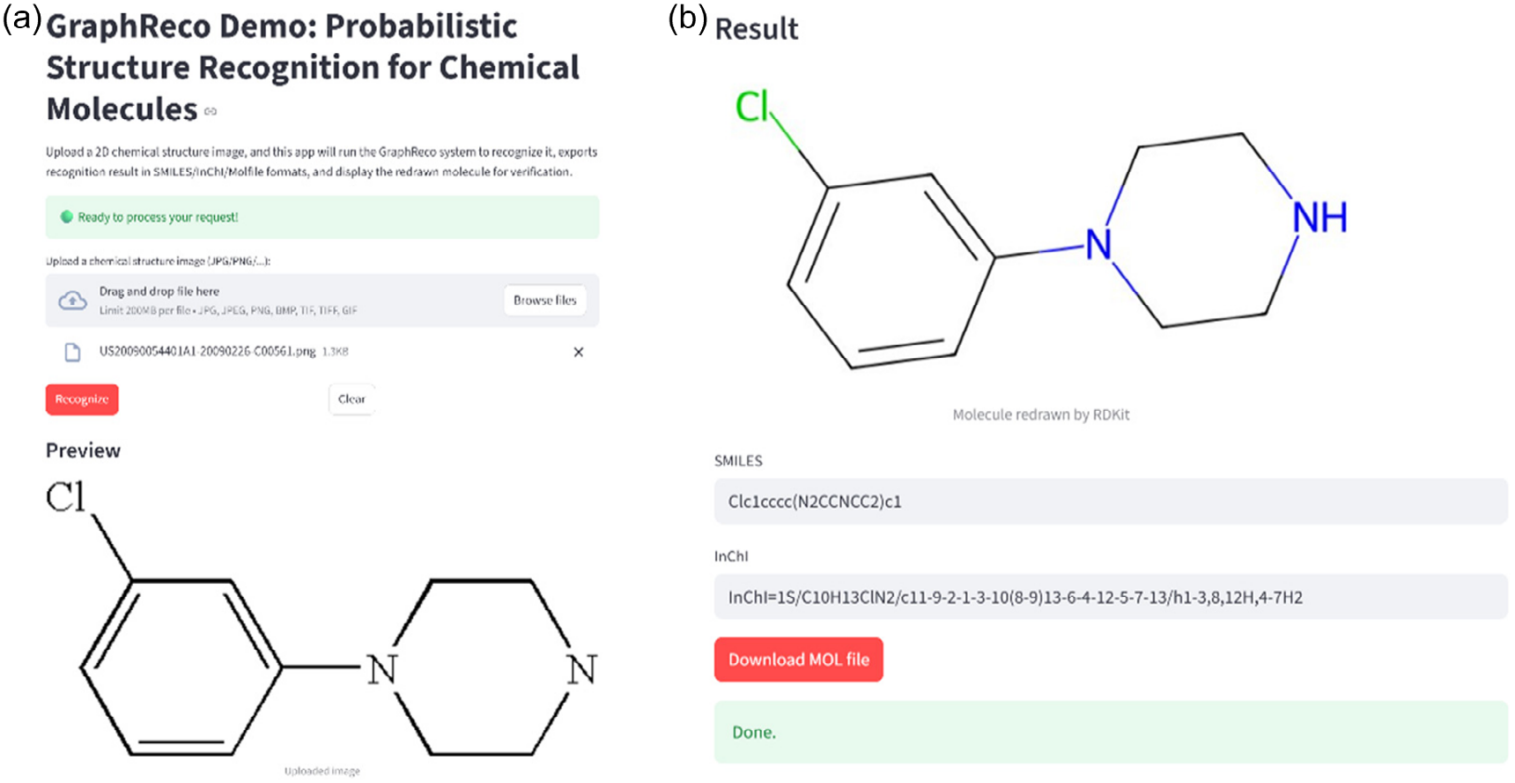
Demo user interface of the GraphReco OCSR system. (a) Chemical structure image upload region and preview of uploaded image. (b) Recognition result presented as redrawn molecule, SMILES string, InChI string, and downloadable Molfile for verification.

We also deployed an online demo (https://graphreco‐demo‐app‐55 700 351 907.us‐central1.run.app/) of our implementation, as shown in Figure 7. The online demo allows users to upload images of chemical structures, then in the backend it will run GraphReco to recognize it and display the redrawn molecule with RDKit. Besides the redrawn molecule, the reconstructed molecule is also available in SMILES, InChI, and Molfile formats for verification and further applications.

We evaluated its recognition performance on standard benchmarks of USPTO‐10K [11], USPTO‐10K‐Abb, and USPTO dataset. The USPTO‐10K, USPTO‐10K‐Abb, and USPTO dataset each contain 10 000, 10 000, and 5719 images of chemical structures and corresponding ground truth Molfiles, all from the United States Patent and Trademark Office (USPTO). In evaluation, the Molfiles in ground truth and exported by GraphReco are converted to International Chemical Identifier (InChI) strings for metrics computation. InChI is a standard string representation for chemical substances, established by International Union of Pure and Applied Chemistry (IUPAC). We use accuracy as the evaluation metric, defined as the percentage of molecules with exactly the same InChI string as ground truth. It measures how many molecules are perfectly recognized. Stereochemistry is removed before conversion to InChI and Markush structures are ignored during evaluation.

### 7.2 Performance Comparison

Table 1 summarizes the performance comparison between our system (GraphReco) and existing ones on benchmark datasets. Compared with other rule‐based and machine‐learning solutions, our system achieves better performance on both the USPTO‐10K and especially the USPTO‐10K‐Abb dataset. On the USPTO dataset, the accuracy of our system is slightly worse than the latest MolGrapher, but better than all other systems. We note that there is a performance gap between older systems (OSRA, MolVec, and Imago) and newer ones (MolGrapher and GraphReco) on the USPTO‐10K‐Abb dataset, but such a large gap is not observed on the USPTO‐10K dataset released together. This suggests that the performance gap is likely caused by the incomplete abbreviation dictionary used in the older systems. In terms of machine learning systems, performance of Img2 Mol on these real‐world datasets is the worst, possibly due to the difficulty of learning not only visual features of structure images but also encoding rules of human‐oriented SMILES notation. On the contrary, MolGrapher leverages the graph model of molecules and reconstructs the molecule graph based on recognition of individual atoms and bonds and thus yields much better performance. From this comparison table we can also know that rule‐based systems, with chemical knowledge integrated, can achieve high recognition accuracy without the need for huge amounts of model training data. And machine learning systems, following the data driven direction, are also catching up with expert‐knowledge‐aided rule‐based solutions on real‐world datasets.

**TABLE 1:** Comparison of chemical structure recognition accuracy between our system and existing OCSR systems on public datasets.*: results from [11].

System	USPTO‐10K (10 000)	USPTO‐10K‐Abb (10 000)	USPTO (5 719)
*Rule‐based systems*			
**GraphReco (ours)**	**94.2**	**86.7**	89.9
OSRA 2.1* [5]	89.7	63.9	89.3
MolVec 0.9.7* [7]	92.4	70.3	89.1
Imago 2.0* [6]	89.9	63.0	89.4
*Machine‐learning systems*			
Img2Mol* [24]	35.4	13.8	25.2
MolGrapher* [11]	93.3	82.8	**91.5**

Bold elements has two meanings: 1) Highlights the name of our system, 2) Highlights the best result on one benchmark among all compared systems. Italic elements are the category name of systems under it, and also tell readers this row is not an actual system being compared.

**FIGURE 8:**
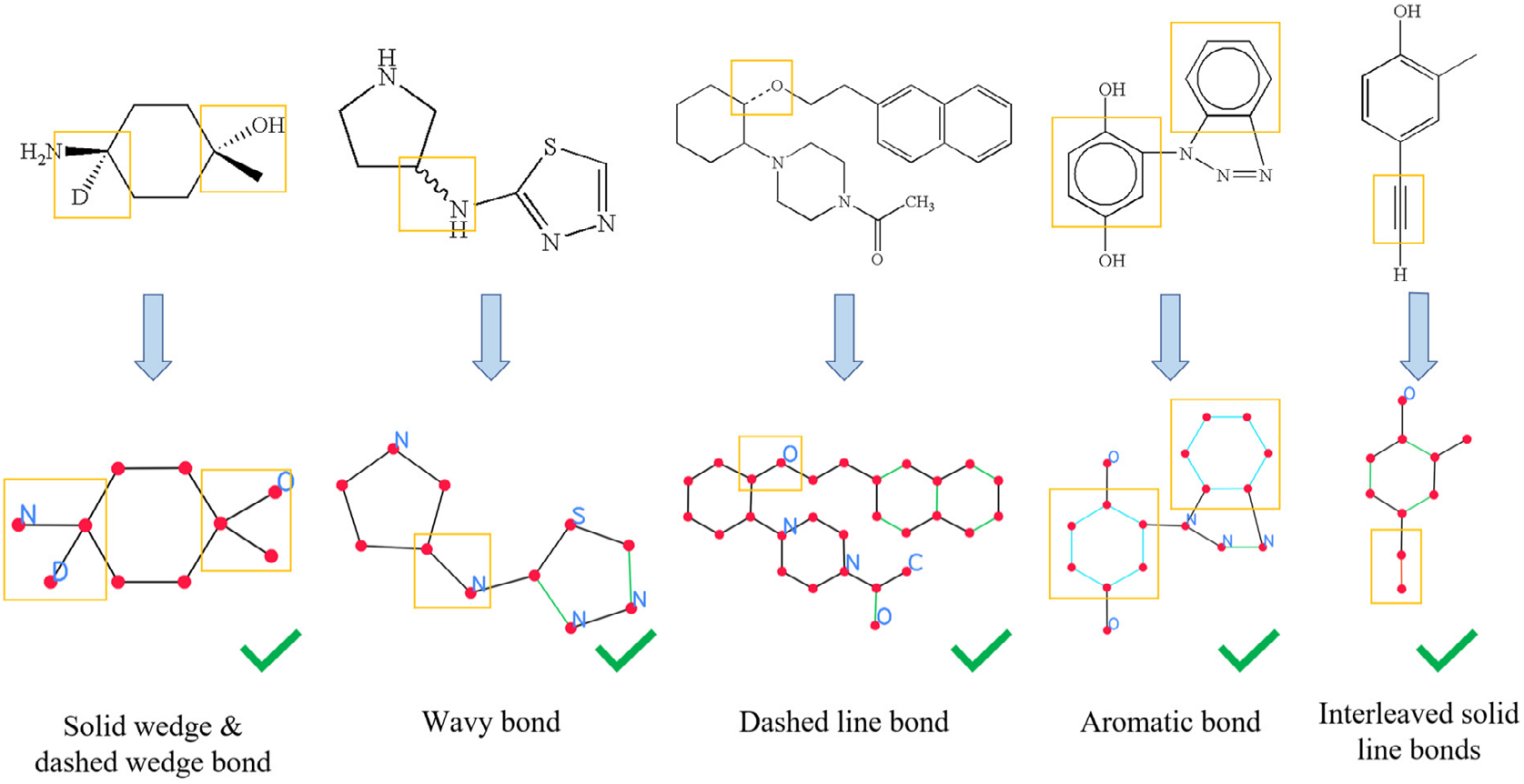
Correct recognition of special substructures. The special substructures are highlighted with yellow boxes. Bonds of different types in the reconstructed graphs are depicted with different colors.

In Figure 8, we show some correct recognition examples of special substructures, including solid wedge bonds, dashed wedge bonds, wavy bonds, dashed line bonds, aromatic bonds, and interleaved solid line bonds. These substructures are less common than the simple solid line bonds, but they are an integral part of molecule structure depictions with their own chemical meanings. Correct recognition of these substructures is essential for the accurate recognition of general molecule structures in the wild.

### 7.3 System Robustness

We evaluate the robustness of our system on a challenging dataset, USPTO‐perturbed, introduced in [24]. Images in USPTO‐perturbed dataset originate from the USPTO dataset, but slight random perturbations are applied, including rotation and shearing, to simulate scanned images. The results are shown in Table 2. From the table, we can see that the performance of OSRA and Imago dropped sharply to below 10%. The accuracy of MolVec, Img2 Mol, and our GraphReco are also within the 30%–40% range. Only MolGrapher stayed relatively high accuracy. The deterministic nature of expert rules in rule‐based systems limit their robustness in case of challenging input. In our system, the probabilistic Markov network is applied to resolve the state ambiguity of atom and bond candidates. Although it yields better performance on the USPTO‐perturbed dataset than other existing rule‐based systems, the accuracy on the challenging dataset is still much lower than the latest data‐driven deep learning system like MolGrapher. This result proves the advantage of recent machine learning OCSR solutions over rule‐based ones on challenging real‐world inputs.

**TABLE 2:** Comparison of recognition accuracy of different systems on perturbed dataset.*: results from [24],^†^: result from [11].

System	USPTO‐perturbed (4 852)
**GraphReco (ours)**	40.6
OSRA 2.1* [5]	6.4
MolVec 0.9.7* [7]	30.7
Imago 2.0* [6]	5.1
Img2Mol* [24]	42.3
MolGrapher^†^ [11]	**86.7**

Bold elements has two meanings: 1) Highlights the name of our system, 2) Highlights the best result on one benchmark among all compared systems.

### 7.4 Ablation Study

To analyze the effect of the FM line detection algorithm, the atom/bond candidate mechanisms, and the Markov network, we turn them off one by one and compare the recognition accuracy on benchmark datasets, as shown in Table 3. When FM line detection is turned off, classical Hough transform is applied to detect bond lines. When atom candidate mechanism is turned off, each bond end is considered an atom, but two atoms are only merged into one when two bonds are connected. When bond candidate mechanism is turned off, one and only one bond between two atoms is considered valid, and bonds that cross atoms are also considered valid. When Markov network is turned off, we use hard thresholds to judge the existence and merging probability of atom/bond candidates.

**TABLE 3:** Performance analysis of FM line detection, atom candidate mechanism, and bond candidate mechanism.

	USPTO‐10K	USPTO‐10K‐Abb	USPTO
GraphReco with full features	**94.2**	**86.7**	**89.9**
GraphReco without FM line detection	2.9	5.5	4.8
GraphReco without atom candidate	9.8	0.4	5.0
GraphReco without bond candidate	79.1	75.8	75.0
GraphReco without Markov network	88.2	81.4	84.2

Bold elements has two meanings: 1) Highlights the name of our system, 2) Highlights the best result on one benchmark among all compared systems.

From Table 3, we can see that without the FM line detection algorithm, recognition accuracy dropped to less than 10% on all of the 3 datasets. This is expected because detection of lines in solid line bonds is the anchor of the whole system. Detected lines are used to identify the most common bond type (solid line bond) and isolate symbols. Inaccurate line detection eliminates the ground for the whole system. The system performance also dropped similarly to less than 10% when atom candidate mechanism is turned off. The atom candidate mechanism works between lower‐level component extraction and final graph assembly. Even though individual bonds and symbols are correctly located, there are still ambiguities in converting them to atoms, as a small deviation in bond to bond/symbol connection may break the correctness of the entire structure. Such ambiguities are effectively eliminated by the resolution process of atom candidate existence and merging. Compared with the atom candidate mechanism, bond candidates have much less impact on the overall performance, indicating that most bonds are correctly identified. The impact of the Markov network is the smallest among the evaluated features. The system can still achieve good results with hard thresholds for candidate ambiguity resolution, but the probabilistic Markov network improves recognition accuracy in occasions where candidates are farther than typical distance.

Table 4 provides some examples of recognition failure when the evaluated features are disabled, and correct recognition results as comparison. From these examples we can have a better sense that the FM line detection algorithm produces better line detection results than classical LHT. Without the atom candidate mechanism, there will be more errors in the determination of connection and unification between atoms. The bond candidate mechanism can fix possible errors in the degree of bonds. And the Markov network helps make the correct decisions on candidate state in ambiguous cases.

**TABLE 4:** Examples of recognition failures when features are disabled and successes when all features are enabled.

	Sample 1	Sample 2	Sample 3	Sample 4
Original image	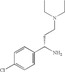	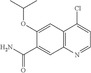	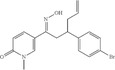	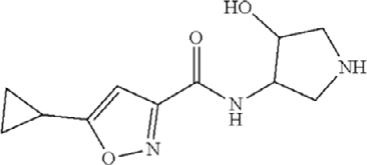
Incorrect result with specific features disabled	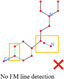	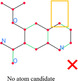	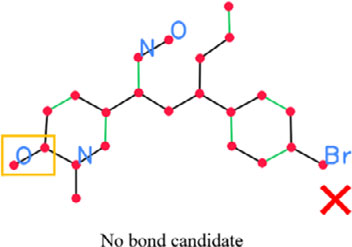	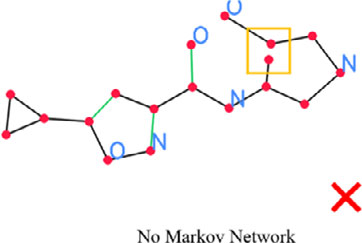
Correct result with full features enabled	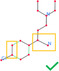	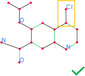	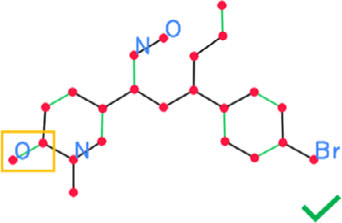	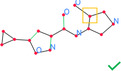

## 8 Conclusion

In this study, we present GraphReco, a knowledge‐driven model, for the recognition of 2D chemical structure images. In GraphReco, we designed an adaptive line detection algorithm to achieve precise detection of bond lines and designed a probabilistic candidate mechanism to resolve decision ambiguities in graph assembly. Evaluation experiments demonstrate that GraphReco achieved outstanding performance on standard benchmarks. This automated recognition solution for chemical structure images can serve as a critical component in publication‐oriented chemical data mining systems. It is expected that digitization of history chemical documents can be facilitated and thus improve productivity in chemical informatics and more fields.

## Conflicts of Interest

The authors declare no conflicts of interest.

## Data Availability

Data sharing is not applicable to this article as no new data were created or analyzed in this study.
